# Contributions of one hypothetical model of predictive relationships between psychological skills and emotional intelligence in university student-athletes: A cross-sectional study

**DOI:** 10.1016/j.heliyon.2023.e19916

**Published:** 2023-09-15

**Authors:** Isabel Mercader-Rubio, Nieves Gutiérrez Ángel, Sónia Brito-Costa, Sofia Silva, Ana Moisão, Guilherme Furtado

**Affiliations:** a Department of Psychology, Faculty of Education Sciences. Universidad de Almería, Spain; b Polytechnic of Coimbra, Coimbra Education School, Research Group in Social and Human Sciences (NICSH), Portugal; c Polytechnic Institute of Porto, Center for Research and Innovation in Education (InED), School of Education, Portugal; d Polytechnic Institute of Coimbra, Applied Research Institute, Portugal; e Polytechnic Institute of Coimbra, Human Potential Development Center (CDPH), Portugal; f Center for Studies on Natural Resources, Environment and Society (CERNAS), Polytechnic Institute of Coimbra, Bencanta, 3045-601 Coimbra, Portugal

**Keywords:** Self-confidence, Positive and negative coping control, Visuo-imaginative control, Attentional control, Attitudinal control, Motivational level

## Abstract

Psychological skills are considered in sport psychology as indispensable capabilities to analyze the athlete's own vision of his or her own personality. These skills encompass self-confidence, positive and negative coping control, attentional control, visual-imaginative control, motivational level, and attitudinal control. The significance of this research lies in demonstrating the relationship established between each of the dimensions of emotional intelligence and the constituent skills of the personality. As such, this study aims to investigate the relationship between the seven factors related to psychological skills and emotional intelligence (attention, clarity, and emotional regulation). The sample comprises 163 university students pursuing degrees in Physical Activity and Sports Sciences, [70,9% (N = 117) men and 27.9% (N = 46) women] with a mean age of 20.33 years. As assessment instruments, we used two validated and standardized scales, the IPED and the TMMS-24. The main findings of this work allow us to affirm the existence of a relationship between the three dimensions of emotional intelligence and the control of both positive and negative coping, attentional control, visual-imaginative control, motivational level, and attitudinal control. In conclusion, this study underscores the necessity of cognitive and emotional training, in addition to physical training, to enhance these skills among both male and female athletes.

## 1 Introduction

Sport psychology contributions attempt to investigate the psychological and behavioral factors of sport development and improvement [1] from a holistic approach centered on the improvement of physical, technical, and tactical components [[2], [3]], [[2], [3]]. It should be clarified that this discipline focuses on different types of athletes: both amateur athletes (those who dedicate their free or leisure time to the practice of a sport), as well as professional athletes (who exercise their profession in sport), or high-performance athletes (who compete in a particular discipline at an international level) [4]. It is precisely in this last profile of the athlete that different psychological variables such as attention, stress, anxiety, self-control, interpersonal skills, self-regulation, moods, cohesion, and emotional adjustment intervene with greater intensity [[5], [6], [7], [8]]. The significance of this research work resides in the recognition of the necessity for not just cognitive and physical training in sports, but also emotional training. As a result, we concentrate on two psychological variables that are particularly relevant for athletes: psychological skills, which are regarded in sports psychology as an essential capacity for analyzing the athlete's vision of his or her own personality (which includes self-confidence, positive and negative coping control, attentional control, visual-imaginative control, motivational level, and attitudinal control), and what influence the different dimensions have on them.

The theoretical basis from which we start to study the main characteristics of the athlete to establish a psychological performance profile is found in the contributions of Loehr [9], who studies and establishes for this purpose seven components that make up the psychological profile of the athlete, which are self-confidence, positive and negative coping control, attentional control, visual control, imaginative control, motivational level, and attitudinal control [[10], [11], [12]]. These contributions led to the idea of investigating athletes' psychological skills, understood [13] as seven components that make up the psychological profile of the athlete, i.e., self-confidence, positive and negative coping control, attentional control, visual-imaginative control, motivational level, and attitudinal control [[14], [15], [16]].

These skills are deemed crucial for analyzing the athlete's own perception of their personality. They are elaborated upon in the following sequence: i) Self-confidence corresponds to the subject's own certainty about his or her abilities, both psychological and physical [[17], [18], [19]]. It is essential that this psychological skill be trained in athletes to be able to cope with the various situations they will have to face, especially when we are talking about athletes who compete at a high level [20]; ii) Coping control, both positive and negative, refers to those strategies that the athlete must use to control attention and motivation. It therefore requires the athlete to have a high level of cognitive and behavioral mastery [21]; iii) Attentional control refers to the ability to focus and sustain attention during sports [22]; iv)Visuo-imaginative control is based on the ability to control the senses and the stimuli perceived during sports [23]; v) Attitudinal control is defined as the ability to sustain routines, habits, and consistency in sports practice [24].

And, subsequently, the motivational level relates to the importance and reasons why the athlete does sport. Such motives can be internal or external [1].

According to the recent literature, the number of research studies related to the analysis of the psychological profile of athletes has been increasing over the years [5,[25], [26], [27], [28]], which have highlighted the fact that high scores in motivation, confidence, attitudinal control, and emotional regulation correspond to athletes with greater psychological skills [[29], [30], [31], [32]], as well as their influence on sporting performance [[33], [34], [35], [36], [37], [38]]. Psychological skills are also currently considered a mediating factor between the different abilities that an athlete possesses (e.g., physical, tactical, and technical) [39], and are even related to the development of sporting talent [40], which allow the athlete to maximize their actions and be effective in the sport practiced [41].

For all these reasons, we consider it essential to take into consideration those aspects of a psychological nature that make up the psychological profile of an athlete, with the aim of improving their performance and sporting practice. However, the importance of studying and researching the psychological profile of the athlete does not stop there, as it is currently considered to be a determining factor when we are talking about athletes in training, a group in which forgetting these issues can lead to high demands and stress [42].

As a theoretical foundation for emotional intelligence, we take the contributions made by the ability models [43], for which emotional intelligence corresponds to a skill that can be taught, learned, improved, and developed. And which in turn is composed of three dimensions: emotional attention, emotional clarity, and emotional regulation. In terms of each of these dimensions, emotional attention is understood as the capacity to contrast and value one's own feelings with those of others. Emotional understanding is known as the ability to explore, catalog, and investigate one's own and others' feelings, and emotional regulation is understood as the ability to collect and deliberate about one's own and others' feelings.

The main contribution of this research is shared by previous studies that, within sport psychology, focused on the influence of emotional intelligence on other types of variables. Among them are those that establish that higher levels of emotional intelligence are directly and positively correlated with self-concept [44],^44^ motivation [[45], [46], [47]], life satisfaction [48], precompetitive anxiety [49,50], basic psychological needs [51], interpersonal relationships in sport [52], identified regulation, introjected regulation, and external regulation in athletes [53], and identified regulation, introjected regulation, and external regulation in athletes [[54], [55], [56], [57]].

Therefore, the main goal of this study is to investigate the relationship between the seven factors related to psychological skills (self-confidence, negative and positive coping control, attentional control, visor-imaginative control, motivational level, and attitudinal control) and emotional intelligence (attention, clarity, and emotional regulation) in university students with degrees related to physical activity and sport sciences. We should point out that in the Spanish university system, there are various studies that enable the teaching of physical education, which are grouped into three areas: official degree studies in physical education and sport sciences. The specific mention of physical education in the official degree studies in primary education Postgraduate or master's degree studies—in this case, the official master's degree in physical activity and sport sciences and the master's degree in teacher training with a mention in physical education — For this research, we chose students belonging to these three categories as a sample. Thus, 76.7% (n = 145) were students of the official bachelor's degree in physical activity and sport sciences or the official bachelor's degree in primary education with a mention in physical education. While 23.3% (n = 18) were students of both the official master's degree in physical activity and sport sciences and the master's degree in teacher training with a mention in physical education.

To substantiate the objective of the study, we examined the following hypotheses.

H1 There is a direct and positive relationship between attention, clarity, emotional regulation, and self-confidence (CT).

H2 There is a direct and positive relationship between attention, clarity, and emotional regulation and Negative Coping Control (CV).

H3 There is a direct and positive relationship between attention, clarity, emotional regulation, and Attentional Control (AC).

H4 There is a direct and positive relationship between attention, clarity, emotional regulation, and visual-imaginative control (CN).

H5 There is a direct and positive relationship between attention, clarity, emotional regulation, and Motivational Level (NM).

H6 There is a direct and positive relationship between attention, clarity, emotional regulation, and Positive Coping Control (CP).

H7 There is a direct and positive relationship between attention, clarity, emotional regulation, and Attitudinal Control (CC).

## 2 Material and methods

### 2.1 Study design and sample

A quantitative, descriptive, and comparative methodology was used. The sampling method employed was basic random. Soper's a priori sample size calculator for structural equation models [58] was used to calculate the sample size. The expected effect size was 0.30, the probability level was 0.05, and the required power level was 0.95, with a minimum recommended effect size of 200 cases. This result implies that the best sample size matched a number close to the total number of participants in our research sample. As inclusion criteria, we considered each participant to be an official student of the course in which the questionnaires were administered, to have signed the informed consent form (the official model of the University of Almeria), and to be of legal age. We eliminated all responses that were not 100% completed or that lacked socio-demographic information. The paper questionnaires were collected, so another exclusion criterion was to check that there was no randomness in the answers or that the answers formed drawings.

### 2.2 Participants

**Fig. 1:**
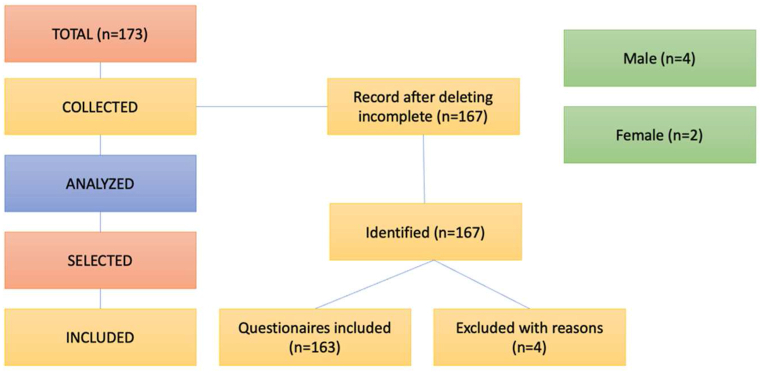
Final sample.

The total number of participants was 173 of whom six were discarded due to incomplete data. Finally, of the 167 final participants, 4 of them were eliminated because errors were detected in completing the questionnaire (Fig. 1). The final sample comprises 163 students: 70.9% (n = 117), and female students: 27.9% (n = 46) with a mean age of 20,33 years (SD = 3.44) related to Physical Activity and Sport Sciences from university degrees, all of them belonging to both official undergraduate and master's degrees (Table 1).

**Table 1:** Description of the sample according to age and sex.

	Females	Males	Total	Under 25 years	Over 25 years
**First course**	18 (39.1)	68 (58.1)	86	86 (97.8%)	2 (2.2%)
**Second course**	16 (34.8)	23 (19,7)	39	38 (97.4)	1 (2.6%)
**Third year**	6 (13%)	14 (12%)	20	20 (100%)	0
**Total**	40	105	145		
**Master's degree**	6 (13%)	12 (10.3)	18	4 (22.3%)	17 (77.7%)
**Total**	46	117	163		

## 3 Ethics statement

This study was approved by the Institutional Review Board of the University of Almería (UALBIO2022/035). The participants who had been previously informed about the study and gave their informed consent, decided to participate in the research. In addition, the entire sample filled out the official informed consent form of the University of Almeria (Spain) and was informed of the data protection protocol.

### 3.1 Instruments

We employed the Trait Meta Mood Scale (TMMS-24) in its Spanish version [59]. This instrument measures self-perceived emotional intelligence (it is a self-report measure) through the different dimensions that make up the construct: attention to feelings, emotional clarity, and emotion regulation, through 24 items using a Likert-type scale (1 = strongly disagree to 5 = strongly agree). The Cronbach's alpha for each subsection is (perception, α = 0.90; clarity, α = 0.90; regulation α = 0.86) and adequate test-retest reliability: perception = α = 0.60; understanding α = 0.70 and regulation α = 0.83 [60]. Specifically, we obtained a Cronbach's alpha α = 0.84.

We also used the Psychological Inventory of Sport Performance (IPED) [12] in its Spanish version [13]. It is composed of 42 items that assess a total of 7 factors, each corresponding to a different psychological skill: Self-Confidence (AC), Negative Coping Control (CAN), Attentional Control (CAT), Visual-Imaginative Control (VIC), Motivational Level (ML), Positive Coping Control (PAC) and Attitudinal Control (ACC) by means of a Likert-type scale (1–5, where 1 meant do not agree at all and 5 meant strongly agree). We obtained an overall Cronbach's alpha (α) = 0.80, and specifically for each of the subgroups, the following scores: Self-Confidence (SC) α = 0.67; Negative Coping Control (NC) α = 0.72; Attentional Control (AC) α = 0.73; Visuo-Imaginative Control (VC) α = 0.66; Motivational Level (ML) α = 0,70; Positive Coping Control (PC) α = 0.73; Attitudinal Control (AC) α = 0.77.

This study was carried out using the SPSS software (version 26), the R statistical analysis tool (version 2015), and the “Lavaan” package.

### 3.2 Procedure

This study is an ex post facto design that meets the condition of including students pursuing degrees in physical activity and sport sciences. The study and design are consistent with a methodological technique generally acknowledged and used in educational and psychological research to investigate participants' levels of agreement and perception on a given topic [61] and was developed according to the following four phases: In phase one, questionnaires were distributed to all students who attended class on the agreed-upon and approved day with the teacher. Following that, in phase two, both the subject teachers and all participants were given information regarding the study's purpose. Prior to completing the questionnaire in phase three, all participants were of legal age, signed the informed permission (official model of the University of Almeria), and were aware of the data protection protocol. Finally, in phase four, the total sample size is conclusive when the total number of students who provided prior informed consent and chose to participate in our research is considered.

### 3.3 Data analysis

After the descriptive analysis was carried out (average, standard deviation, and bivariate correlations), the reliability analysis and the modeling of second-order structural equations were followed. (SEM). To support the proposed model, the following indices were considered: TLI (Tucker-Lewis Index), SRMR (Standardized Root Square Average Residues), and RMSEA (root mean square error of approximation) [[62], [63], [64]]. The reason for using SEM is that it corresponds to a more well-known comparative analysis test. This method allows us to estimate a data set and introduce a better estimator in this set, which gives us the opportunity to extend the research. The data are consistent with the normality assumption, and for bivariate correlations, the Pearson coefficient was considered.

## 4 Results

Table 2 shows the relationships between each of the dimensions of emotional intelligence (AE/CE/RE) and their relationship with the psychological skills (Self-Confidence (CA); Negative Coping Control (NC); Attentional Control (AC); Visual-Imaginative Control (VC); Motivational Level (ML); Positive Coping Control (PC); Attitudinal Control (AC), measured by more than one dimension (Emotional Attention (EA); Emotional Clarity (EC); and Emotional Regulation (ER).

**Table 2:** Bivariate correlation between each of the dimensions of emotional intelligence and psychological skills.

	AEM	CE	RE	AC	CN	CT	CV	NM	CP	CC
**AEM**				,209**	,141	,183*	,276**	,331**	,179*	,249**
**CE**				,061	,210**	,084	,036	,112	,083	,221**
**RE**				,049	,157*	,059	,128	,175*	,133	,308**
**AC**					,305**	,234**	,513**	,561**	,620**	,645**
**CN**						,223**	,276**	,285**	,150	,145
**CT**							,315**	,225**	,173*	,251**
**CV**								,614**	,664**	,548**
**NM**									,651**	,655**
**CP**										,753**
**CC**										

Note. *p < .05; **p < .01. AEM: emotional care; EC: emotional clarity; ER: emotional regulation; AC: self-confidence; NC: Negative Coping Control; TC: Attentional Control; CV: Visual-Imaginative Control; NM: Motivational Level; PC: Positive Coping Control; AC: Attitudinal Control.

The correlations were positive and reflected the study variables' reciprocity. The information in Table 2 was obtained from the bivariate correlation scores. The closer they are to +1, the higher their association. An exact value of +1 would indicate a perfect positive linear relationship. And in this case, the variables would be associated in a direct sense; the correlations between the variables were positive, reflecting the reciprocity between the study variables.

### 4.1 Structural equation model

**Fig. 2:**
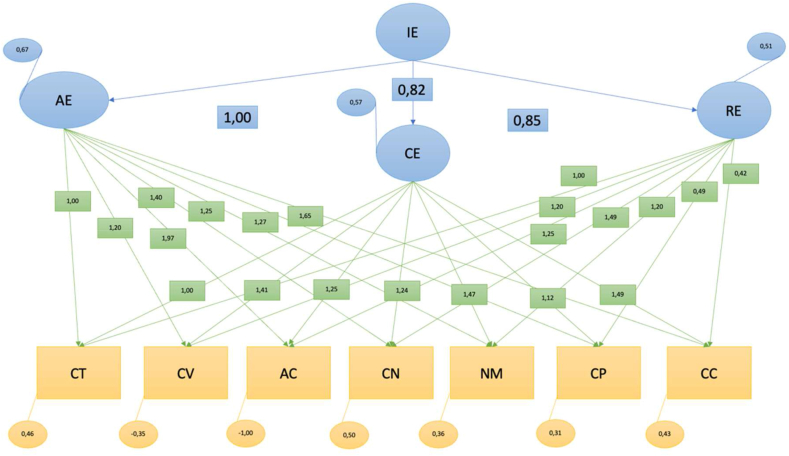
Structural equational model. Legend: EI: Emotional Intelligence; EO: Emotional Attention; EQ: Emotional Clarity; RE: Emotional Regulation; CT: Self-confidence; CV: Negative Coping Control; AC: Attentional Control; CN: Visual-Imaginative Control; NM: Motivational Level; CP: Positive Coping Control; CC: Attitudinal Control.

Fig. 2 depicts a hypothetical predictive model, illustrating relationships through the provided indicators. The comprehensive indicators for model evaluation meet satisfactory standards, as evidenced by a significance level surpassing 0.05, with a statistically significant outcome of p < .001. The Root Mean Square Error of Approximation (RMSEA), a measure assessing model fit, demonstrates a commendable value of 0.02, well below the threshold of 0.08, signifying a favorable fit. Additionally, the Goodness of Fit Index (GFI), which ranges from 0 (indicating poor fit) to 1 (denoting perfect fit), attains an impressive value of 0.98. This GFI value surpasses the threshold of 0.90, further confirming the model's commendable fit to the data [[62], [63], [64]].

Comparing the proposed model with the model of independence or absence of relationship between the variables (Incremental or comparative fit indicators): Non-Normalized Fit Index (NNFI) or Tucker-Lewis Index (TLI): This index tends to 1 for models with a very good fit, with values above 0.90 being considered acceptable, although values above 0.95 would be ideal. We obtained NNFI = 0.99 and TLI = 0.99. Comparative Fit Index (CFI): indicates good model fit for values close to 1, and values above 0.95 are recommended. We obtained a CFI of 0.99. Incremental Fit Index (IFI): Values close to 1 are considered acceptable, especially values greater than 0.95. We obtained an IFI of 0.99 [[62], [63], [64]].

Assessing the quality of the model fit in terms of the number of coefficients estimated to achieve this level of fit (Parsimony indices): Adjusted Goodness of Fit Index (AGFI): Values above 0.90 are indicative of a good fit of the model to the data. We obtained AGFI = 0.94 [64].

The correlation coefficients were used to verify the strength of the relationship between two variables. A correlation coefficient greater than zero indicates that there is a positive relationship, while a value less than zero indicates a negative relationship, and a value of zero indicates no relationship between the two variables being compared. The closer the value of ρ is to +1, the stronger the linear relationship. The relationships established in the structural equation model show that there is no direct and positive correlation between emotional intelligence and self-confidence; Emotional intelligence and negative coping control were positively correlated (=0.041, p .001); Emotional intelligence and attentional control were positively correlated (=0.056, p .001); and Emotional intelligence and visuo-imaginative control were also positively correlated (=0.053, p .001); The primary outcomes of this study substantiate a significant relationship between the three dimensions of emotional intelligence and the control of both positive and negative coping. This implies that athletes exhibiting heightened emotional intelligence levels demonstrate a more proficient utilization and regulation of motivation. In essence, their cognitive and behavioral domains are more effectively orchestrated. Furthermore, a prominent discovery within our findings is the substantial capacity of individuals to enhance focus and sustain attention during sports engagement.

Another noteworthy discovery is the alignment between the three facets of emotional intelligence and visual-imaginative control. This suggests that athletes boasting elevated levels of emotional intelligence exhibit an enhanced aptitude for governing sensory perceptions and stimuli during sports participation. Lastly, our results reveal that athletes endowed with elevated emotional intelligence levels display an increased propensity to uphold routines, establish habits, and maintain steadfast dedication to their sports practice.

In this way, our results show that the emotional ability related to the ability to explore, catalog, and investigate one's own feelings and those of others is closely related to coping control, both positive and negative, and attentional and attitudinal control. As is the case with emotional ability related to the ability to collect and deliberate on one's own feelings and those of others. In other words, athletes with high levels of clarity and emotional regulation control motivation better, have greater cognitive and behavioral domains, better focus and maintain their attention, senses, and stimuli during sports practice, and have a greater ability to maintain routines, habits, and consistency in sports practice.

In order to take into account variables such as sex or age, we performed the linear mediation test to find out whether the variable sex or the variable age affected another variable [65], analyzing the effect of sex on the dependent variable. (See Table 2). In the case of sex, the results obtained indicate that it did not act as a mediating variable between emotional intelligence and psychological abilities. However, in the case of age, it did act as a mediating variable on Self-confidence (p = .00); Negative Coping Control (p = .00); Attentional Control (p = .01); Visual-imaginative control (p = .00). The Motivational Level (p = .00); Positive Coping Control (p = .01); Attitudinal Control (p = .01).

## 5 Discussion

The main objective of this study was to examine the relationship between psychological skills and emotional intelligence in sports and physical activity science students. As a result, the objective of the research has been achieved. Regarding the hypotheses raised in our research, with respect to H1 (there is a direct and positive relationship between attention, clarity, emotional regulation, and self-confidence (TC)), this hypothesis is not fulfilled. Our findings do not suggest the existence of a direct and positive relationship between attention, clarity, regulation, and self-confidence. In this regard, our findings contradict earlier scientific contributions demonstrating such a direct and beneficial association [14].

Regarding H2 (there is a direct and positive relationship between attention, clarity and emotional regulation and Negative Coping Control (CV)), our results indicate that this hypothesis is fulfilled. Therefore, a person with the ability to explore, catalog and investigate their own feelings and those of others, to collect and deliberate on their own feelings and those of others, better copes with adverse situations. The same is the case with the H3 (there is a direct and positive relationship between attention, clarity, emotional regulation, and Attentional Control (AC)) and H6 (there is a direct and positive relationship between attention, clarity, emotional regulation, and Positive Coping Control (CP)). The findings obtained indicate that this hypothesis is fulfilled and that athletes with high levels of emotional intelligence correspond to people who better focus and maintain their attention, senses, and stimuli during sports practice. As for H4 (there is a direct and positive relationship between attention, clarity, emotional regulation, and visual-imaginative control (CN)), our findings indicate that this hypothesis is fulfilled, and that greater cognitive and behavioral domains correlate directly and positively with emotional intelligence. Regarding H5 (there is a direct and positive relationship between attention, clarity, emotional regulation, and the Motivational Level (ML)), the hypothesis is fulfilled since our findings show that emotional intelligence has a direct relationship with the ability to maintain routines, habits, and consistency in sports practice. Finally, and regarding H7 (there is a direct and positive relationship between attention, clarity, emotional regulation, and Attitudinal Control (CC)), our results indicate that this hypothesis is also fulfilled. For all these reasons, the fulfillment of our hypotheses confirms that there is a direct and positive relationship between emotional intelligence (and its three dimensions: attention, clarity, and emotional regulation) and psychological skills related to negative coping control, attentional control, visual-imaginative control, motivational level, positive coping control, and attitudinal control [[66], [67], [68], [69], [70]].

### 5.1 Linking emotional intelligence to psychological control

The main contribution of this study lies in the demonstration that athletes with adequate levels of emotional intelligence will have better cognitive and behavioral domains, a greater ability to focus and maintain attention during sports practice, and a better ability to control the senses and the stimuli perceived during sports practice. As well as to maintain routines, habits, and constancy in sports practice. Several publications support the idea that sport psychology currently enjoys a growing number of publications [8]. In view of this, we can affirm the importance of all those issues related to the study and analysis of the factors that affect the psychological capacities of the athlete and his or her performance, which are becoming increasingly important. Considering this proliferation in the number of research studies, we would also like to highlight the importance of the figure of the sports psychologist, as well as the importance not only of physical training but also of mental training.

This research is in the same field as other similar studies, whose main objective has been to investigate the psychological skills associated with sports performance in amateur athletes of different disciplines [[71], [72], [73], [74], [75]], in referees [[73], [74], [75]], or elite athletes [76]. Many investigations have focused on psychological variables such as sport anxiety [77], flow [78], or injury etiology [79], however, none of them has concentrated on the correlations that can be built between psychological qualities related to athletic performance and emotional intelligence [[80], [81], [82], [83], [84]]. As a result, this is one of the primary contributors to the development of emotional intelligence.

### 5.2 Enhancing performance through emotional intelligence

The findings of this study accentuate the potential of emotional intelligence as a pivotal tool in optimizing sports training and performance outcomes. The demonstrated positive correlation between emotional intelligence and negative coping control highlights emotional intelligence as a potential buffer against the negative impact of stressors on athletes' performance [85]. Moreover, the positive associations between emotional intelligence and attentional control, visuo-imaginative control, and motivational level unveil avenues for targeted training interventions that harness emotional intelligence to augment athletes' attentional focus, sensory perception, and intrinsic motivation [52].

## 6 Implications for sports training

The implications stemming from this study hold tangible value for sports training and coaching methodologies. The recognition that emotional intelligence can significantly impact coping strategies suggests the incorporation of emotional skill development into training programs [86,87] aiding athletes in effectively managing challenges and adversities. The newfound understanding of emotional intelligence's influence on attentional focus and visual-imaginative control offers an innovative angle for enhancing individuals' mental imagery and concentration [88], ultimately contributing to more informed decision-making during competitions.

### 6.1 Fostering athlete consistency and habits

One of the most intriguing revelations stemming from this study pertains to the observed positive correlation between emotional intelligence and attitudinal control. This correlation accentuates the significance of emotional intelligence as a pivotal catalyst in nurturing and sustaining consistent routines and habits among athletes [89]. This critical insight highlights the indispensable role of emotional skills in cultivating enduring discipline and unwavering dedication – attributes that stand as paramount prerequisites for attaining and sustaining success within the fiercely competitive landscape of sports. The profound implication of this finding underscores the potential to strategically integrate emotional intelligence development within athlete training regimens [90], not only to optimize performance in the immediate context but to also fortify the bedrock for consistent, long-term achievement. In essence, this revelation elucidates a pathway to forge individuals’ excellence that extends beyond sporadic triumphs [91], emphasizing the transformative impact of emotional intelligence on fostering a resilient and unwavering foundation of consistency and virtuous habits.

### 6.2 Strengths and limitations

The study's strengths lie in its comprehensive exploration of the intricate relationships between emotional intelligence dimensions and a diverse range of psychological controls, offering valuable insights into the multifaceted impact of emotional intelligence on athletes' cognitive and behavioral capacities. In terms of limitations, a degree of caution is warranted when interpreting these findings, primarily stemming from the relatively modest participant count within the sample, constituting one of the study's inherent weaknesses. Furthermore, given the cross-sectional nature of this research, the scope for establishing causal relationships is inherently limited. Thus, in order to substantiate and strengthen the robustness of these outcomes, a replication of this study with a larger and more diverse participant pool becomes imperative. Addressing the notable limitation of sample size stands as a significant step towards enhancing the credibility of our study's conclusions.

### 6.3 Perspective for future studies and practical implications

Future investigations should delve into potential divergences associated with the specific type of sport engaged in, as well as the unique characteristics inherent to each sport. This avenue of exploration, as previously undertaken in prior studies, can provide nuanced insights into how these psychological attributes might vary across different sporting contexts [[92], [93], [94]]. Future research should also focus on gender and age disparities, as well as their influence and variations in the links between psychological skills and emotional intelligence. Moreover, an appealing trajectory for forthcoming research involves adopting a longitudinal framework. Such an approach, spanning the duration of the initial university program, can offer a dynamic perspective on the trajectory of these psychological attributes. This extended observation period has the potential to unravel the temporal dynamics, elucidating the evolution and stability of the identified relationships over time. This could notably enhance our understanding of how emotional intelligence interacts with various psychological controls across the span of a student-athlete's educational journey.

## 7 Conclusion

In conclusion, the present study offers substantiating evidence for the anticipated relationships between the three dimensions of emotional intelligence and the regulation of positive and negative coping mechanisms, attentional control, visual-imaginative mastery, motivational levels, and attitudinal command within the constructed models. Within this context, emotional intelligence emerges as a pivotal element contributing to the enhancement of these proficiencies among athletes. Consequently, emphasizing emotional training becomes paramount for aspiring individuals in the field of physical activity and sport sciences right from the outset, equipping them to adeptly navigate various sporting and professional scenarios.

## Declarations

Ethics statement approval and consent to participate: The study was approved by the Institutional Review Board of University of Almería (UALBIO2022/035) and was performed in accordance with the Declaration of Helsinki. The participants have been previously informed about the study, decided to participate in the research and gave their written informed consent. In addition, the entire sample filled in the official informed consent form of the University of Almeria (Spain) and was informed about the data protection protocol.

## Availability of data and material (ADM)

The datasets used and/or analyzed during the current study are available from the corresponding author on reasonable request.

## Funding

This research received no external funding

## Author contributions

The inception and formulation of this study's concepts and methodologies were collaboratively undertaken by I.M.-R., S.B.C., and N.G.Á. The software aspect was meticulously managed by S.S. and S.B.C., while the validation process encompassed contributions from I.M.-R., N.G.Á., and S·B.C. N.G.Á. played a pivotal role in conducting formal analyses, unraveling key insights. The investigation phase was an orchestrated effort involving I.M.-R., N.G.Á., S·S., G.E.F., and S.B.C., collectively navigating through data and findings. The allocation of resources was a joint endeavor led by I.M.-R., S.B.C., and N.G.Á. Data curation was skillfully executed by I.M.-R., N.G.Á. and, S·B.C. ensuring precision and reliability. Visualization of outcomes was collectively achieved by I.M.-R., N.G.Á., and S.B.C., capturing the essence of the study's results. In terms of supervision, I.M.-R., N.G.Á., and S·B.C. provided guidance and oversight throughout the study. The administration of the project was skillfully managed by I.M.-R., N.G.Á., S·S., A.M., G.E.F., and S.B.C., orchestrating the logistical aspects seamlessly. The foundational draft of the study was carefully composed by N.G.Á., I.M.R., and S.B.C., laying the groundwork for subsequent iterations. The refinement of the manuscript thorough last review and editing was a collaborative effort between S.B.C., G.E.F and N.G.A. This collective effort culminated in a comprehensive and robust research endeavor.

## Declaration of competing interest

The authors declare that they have no known competing financial interests or personal relationships that could have appeared to influence the work reported in this paper.
